# Updates on Plant-Based Protein Products as an Alternative to Animal Protein: Technology, Properties, and Their Health Benefits

**DOI:** 10.3390/molecules28104016

**Published:** 2023-05-11

**Authors:** Xiao Xiao, Peng-Ren Zou, Fei Hu, Wen Zhu, Zhao-Jun Wei

**Affiliations:** 1 College of Animal Science and Technology, Anhui Agricultural University, Hefei 230036, China; xiaox@stu.ahau.edu.cn; 2 School of Food and Biological Engineering, Hefei University of Technology, Hefei 230601, China; 2020111398@mail.hfut.edu.cn (P.-R.Z.); hufei@hfut.edu.cn (F.H.)

**Keywords:** plant-based protein, alternative protein, technology, sensory properties, health benefits

## Abstract

Plant-based protein products, represented by “plant meat”, are gaining more and more popularity as an alternative to animal proteins. In the present review, we aimed to update the current status of research and industrial growth of plant-based protein products, including plant-based meat, plant-based eggs, plant-based dairy products, and plant-based protein emulsion foods. Moreover, the common processing technology of plant-based protein products and its principles, as well as the emerging strategies, are given equal importance. The knowledge gap between the use of plant proteins and animal proteins is also described, such as poor functional properties, insufficient texture, low protein biomass, allergens, and off-flavors, etc. Furthermore, the nutritional and health benefits of plant-based protein products are highlighted. Lately, researchers are committed to exploring novel plant protein resources and high-quality proteins with enhanced properties through the latest scientific and technological interventions, including physical, chemical, enzyme, fermentation, germination, and protein interaction technology.

## 1. Introduction

With the rise in consumers’ demands, animal proteins are gradually in short supply, which has led to a trend towards seeking alternatives to animal proteins. In addition, with the evolving focus on nutrition and health, consumption patterns have changed towards differentiation, individualization, and simplification. When it comes to protein consumption, people are considering a number of future-oriented social issues such as health, safety, environmental pollution, and resource availability [1]. In terms of protein-rich food, the nutritional and health values of plant and animal proteins are different. Although animal protein has a high nutritional value, the intake of some animal protein (especially fatty meats) is often accompanied by a high intake of saturated fat [2]. An excessive intake of animal fat can increase the risk of chronic diseases such as hypertension and hyperlipidemia [3]. However, plant proteins are nutritionally comprehensive, similar to animal proteins, easily digested and absorbed by the body, and have a variety of physiological health functions [4]. In addition, the spread of plant-based protein products can reduce the need to feed animals and slaughter them and plant-based farming can reduce the land area and carbon emissions and lower water consumption compared with animal farming, resulting in environmental sustainability and resource conservation [5]. With a common consideration for both, plant-based foods are highly favored for their attributes of preservation of environmental resources and their related benefits to human health [6]. With the preferred consumption of plant-based proteins, the incidence of diet-related health problems such as obesity and diabetes can be greatly reduced. Furthermore, plant-based proteins can supplement preventive effects on cancer and cardiovascular diseases [7]. There are already some plant protein products on the market that have replaced animal protein products in terms of sensory, nutritional, and functional benefits. Therefore, more eminent steps in this budding area of research would be to address the human demand for animal protein products and the pressure on environmental resources caused by traditional farming, to reduce the health problems caused by the excessive intake of animal protein, to improve consumers’ health, and to improve the development of plant-origin proteins [8].

Although plant-based protein foods have been accepted by consumers, there is still scope for development and their future commercial market potential should not be underestimated [9]. Among plant-based foods, the largest category (and the most developed) is none other than plant milk. Moreover, plant-based milk had a penetration rate of 39% in US households, which accounted for 15% of sales in the milk category. Plant-based meat is the second largest plant-based product, with the Asia-Pacific market growing the fastest and Europe becoming the largest market. Plant-based eggs are the fastest growing category in the plant-based segment. It is evident that the development of new plant-based protein products has gradually become a hot topic of research in the food industry and a range of products have been developed, such as plant-based protein emulsion foods [10]. A number of food companies in developed countries in Europe as well as the United States have already been marketing plant-based proteins in advance and have not only received significant venture capital investment but have also had many core patent technologies and have launched a number of products [11]. With the rapid development of bioscience and food technology, more and more plant protein products are being studied and developed as alternatives to animal proteins.

The objective of this updated review is to explore the latest types of plant-based protein products as an alternative to animal protein, evolving processing technologies, functional properties, and their health benefits. Based on common and popular plant-based protein products on the market, we present the types of animal protein alternatives, mainly concentrating on four categories: plant-based meat, plant-based eggs, plant-based dairy products, and plant-based protein emulsion foods. At present, there is a large gap between the use of plant proteins and animal proteins, leading to some challenges such as poor functional properties, insufficient texture, low protein biomass, allergens, and off-flavors, etc. Thus, this review provides updated information on the main methods and techniques to improve the functional properties of plant proteins and to optimize the source and quality of plant proteins. We explore the possible solutions to address the existence of obstacles and constraints for plant-based alternative proteins, which not only contribute to the development of plant-based protein but also strengthen its economical and accessible nutritional value in the food and health industries. Moreover, we also highlight the properties and nutritional and health benefits of plant-based protein products.

## 2. Types of Plant-Based Protein Products as Alternatives to Animal Proteins

### 2.1. Plant-Based Protein Meat

**Figure 1:**
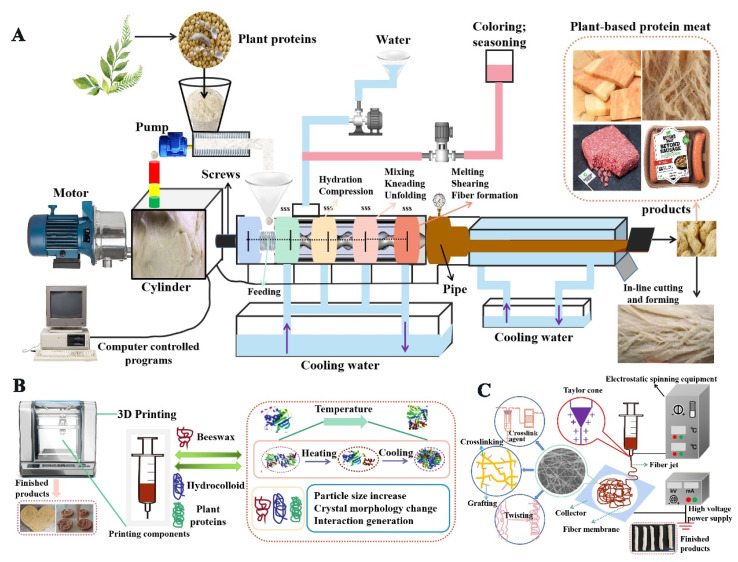
Representation of detailed processing techniques for plant-based protein meat. (**A**) Double-screw high-moisture extrusion; (**B**) 3D-printable; (**C**) wet-spinning technique.

With the increasing demand for meat, environmental requirements, and health needs, there is an alternative market base for plant-based protein meat, with the number of commercially available products increasing year by year that are widely recognized by consumers [12]. Plant-based protein meat is a collective term for food products with a similar flavor, texture, and form to animal meat products, made from plant-based raw materials (e.g., cereals, pulses, etc., and algae, fungi, etc.) as the main source of protein. Among them, it is mainly produced through high-moisture extrusion, 3D printing, and electrostatic spinning technologies, of which the working principles are shown in Figure 1. The main characteristic of plant-based protein meat is that it has a fibrous or drawn structure very similar to that of animal meat; this structure owes its formation to the organization of the protein. In addition, the process of plant-protein histochemistry can be viewed similarly as an irreversible protein thermo–gel reaction involving changes in the secondary and tertiary structure of protein molecules, but it rarely involves transformations in the primary structure [13]. Extrusion technology is a continuous heat treatment process, which is widely used in the production process of plant-protein meat due to its advantages such as short-time consumption, high efficiency, low cost, and mature technology. Under the combined effect of temperature, pressure, and shear, the material is mixed, homogenized, extruded, expanded, and shaped to form a fibrous structure of organized protein (Figure 1A). Electrostatic spinning is a process in which plant proteins are denatured by being dissolved in alkaline reagents and then coagulated through a spinneret to form a fibrous material, resulting in organized proteins (Figure 1C). Three-dimensional printing is a new processing method combined with extrusion, where the molten protein is solidified by extrusion through a spinneret and covered by layers, thus forming a fibrous structure (Figure 1B). In addition to the above techniques, colloidal structure-design techniques such as directional freezing, directional stretching, and double network gels can also be applied to the manufacturing of plant-based artificial meat by simulating the multi-level anisotropic structural characteristics of real meat [14]. The structure of the obtained organized plant protein is still somewhat different from that of real meat due to technological limitations and the molecular structure of plant proteins [15].

The plant-based alternative proteins currently used in commercially available plant-based meat products are mainly soy, pea, and wheat proteins, followed by potato, rice, and mung bean proteins [14]. Soy protein, the most widely used raw material, can promote the formation of fibrous meshwork due to its high content of sulfur-containing amino acids in the globulin molecular chain, which can easily open the chain when subjected to thermal shear, exposing the molecular binding sites and further oxidation to generate disulfide bonds [16]. According to Xiang et al. (2022) [17] and Hu et al. (2022) [18], wheat gluten protein contains two types of proteins that promote the orientation of protein fibrillation, namely glutenin and gliadin. Under high-moisture extrusion conditions, the low-protein content can also achieve fibrosis by interacting with wheat gluten proteins. This is due to the fact that wheat gluten protein has a stronger ability to unwind its molecular chains and easily bind to other molecules compared with other proteins and can generate solid disulfide bonds, which in turn promote protein rearrangement and the formation of fibrous structures with a weblike structure [19]. Meanwhile, studies have been reported on wheat gluten in the field of extrusion histochemistry, suggesting that the addition of gluten improves the fibrous reticulation of the protein and helps to improve the quality of the extruded product [18,20,21]. However, wheat gluten may not be suitable as a raw material alone for extrusion organizers, because when wheat gluten combines with water it tends to form a mesh structure [19]. From a processing point of view, products produced from a single protein fraction tend to be lacking in processing qualities (water-holding, oil-holding, ductility, etc.), so there is a tendency to mix multiple protein fractions, such as soy–peanut, wheat–soy, soy–pea, etc. [22]. Peanut protein, as a raw material protein, compared with soy protein, contains less antinutrients, high-soluble protein, and nitrogen-solubility index and is easily accepted and digested by the human body. In recent years, pea protein has been increasingly studied in extrusion organization due to the fact that pea protein is comparable with and complementary to soy protein [22]. Although pea protein is inferior to soy protein in terms of thermal gelation properties, it is still considered an excellent alternative to soy protein, mainly because the addition of salt ions during processing and improved processing conditions can enhance the stability between the molecular forces of pea protein and improve its gelation properties [23]. Plant proteins, as raw proteins for plant-based artificial meat, currently have relatively few protein types that can be used in production. A large number of studies have been conducted on the preparation of plant proteins for various plant species [24]. However, they are only in the laboratory stage and cannot be implemented for effective commercial application due to the limited production technology as well as the narrow scale of industrialization, which also includes many factors such as raw material sources, operating costs, and processing difficulties. Therefore, advanced processing technologies and optimized production equipment are needed to reduce the high raw-material requirements and lower production costs and to advance the commercialization of more plant-based proteins [25]. It is inevitable that protein complex systems for plant-based artificial meat components will become more diverse and the number of commercially available products will increase, which will increase the opportunities for the promotion of plant-based meat in the future.

### 2.2. Plant-Based Milk

Plant-based milk is considered an alternative to milk for personal health reasons, especially for some people with metabolic disorders and allergies, for example, lactose intolerance (LI) and milk protein allergy (CMPA) [26]. In recent years, the demand for plant-based milk has been increasing due to its benefits, including lower production costs, hypo-allergenicity, unique technical functional properties, and a shift in consumer preference towards plant-based diets. From a nutritional point of view, plant-based milk has a lower protein, calcium, and vitamin D content than animal milk and it does not contain the full range of essential amino acids (other than soy milk) [26]. However, it has unique nutritional advantages, such as the presence of unsaturated fatty acids (linoleic, α-linolenic acids, etc.), dietary fiber, and some plant secondary metabolites, making it possible to replace animal milk [27]. Plant-based milk has a positive effect because of its rich antioxidant activity and fatty acids that can reduce the risk of cardiovascular disease, cancer, atherosclerosis, and diabetes. Common plant-based milks can be broadly classified into four categories: soy milks, nut milks (represented by almond and walnut milks), grain milks, mainly oat and quinoa milks, and vegetable and fruit milks such as coconut milk. There are five categories based on the ingredients used in milk substitutes: legumes (chickpeas, soya), nuts (almonds, Brazil nuts, cashews, hazelnuts), cereals (oats, rice), seeds (sesame, sunflower), and pseudo-quinoa (quinoa) [28]. There are still some bottlenecks regarding development, such as the presence of antinutritional factors (trypsin inhibitor, phytic acid, and saponin), the homogenization of product categories, and the stability of products [27]. In view of this, it is particularly important to adopt advanced processing technologies to improve the quality of the final product.

Plant-based milk refers to products made from plants and/or their products that contain certain proteins as the main raw material, to which food additives and food supplements can be added. Plant-based proteins have different nutritional values, so fortifying them by adding enzymes or blending two or more plant proteins to achieve a product with the same high nutritional value as milk can be an important step in processing [27]. The addition of salt and sweeteners is used to develop organoleptic properties and gums are adopted to improve stability. Vitamin and mineral enrichment is also a crucial issue for consumers who prefer plant-based milk alternatives. The low bioavailability of vitamins and minerals for plant-based milk due to some antinutrients and polyphenols can be overcome by fermentation, sprouting, chelating agents, exogenous phytase, or heat treatment [29]. Most of the plant-based milks available on the market are made from plant seeds, nuts, and pulp, which are processed by reducing the size of the raw material, including a series of steps such as soaking, crushing, grinding, blending, homogenizing, and sterilizing. Among them, the use of homogenization and heat treatment processes is essential to improve the suspension and microbiological stability of the final product [28]. The main problems in the production of plant protein milk are the extraction rate of proteins, the stability of emulsified protein drinks (flocculent precipitation, fat precipitation, and browning), shelf-life, the removal of physiologically harmful factors (such as soybean trypsin inhibition factor, cyanogenic hydrogen acid in bitter almonds, etc.), and the occurrence of off-flavors (such as beany taste, bitterness, etc.), which need to rely on process technology, equipment, and formulations [28]. In some cases, new technologies such as ultrasound, pulsed electric fields, ohmic heating, and ultra-high-pressure homogenization have been applied to improve their stability without the use of additives [30]. Bocker and Silva (2022) showed that the homogenization process reduced the particle size in almond beverages but produced unstable emulsions and phase separation. When a high pressure at 300 °C was combined with the homogenization process, the physical stability and appearance of almond beverages during storage was improved [31]. This is due to the correlation between the solubilization of the proteins and the subsequent denaturation of the emulsions during heat treatment, which helps to stabilize particle dispersion and avoid phase separation, resulting in a more stable emulsion. Salve et al. (2019) subjected freshly extracted peanut milk to different intensities of sonication to improve the final quality of milk [32]. This was due to the high energy density and duration of the treatment, which increased the microbial inactivation capacity and breakdown of the peanut cells, leading to the increase of the hydrolyzed protein content and a better settling index to avoid phase separation. In addition, the microstructure was improved by smaller particle and fat globule sizes. New thermal emerging technologies with nonthermal processing techniques were investigated to address issues such as extended shelf-life without the use of high temperatures, as well as emulsion stability, nutritional integrity, and organoleptic acceptability [31]. The emergence of plant-based protein milks has provided consumers with a wide range of nutritional options. As a member of the animal protein alternatives, there are still some bottlenecks that hinder its development, such as the presence of antinutritional factors and the homogeneity of the product range. In view of this, it is important that advanced processing technologies are applied to improve the quality of plant-based milk.

### 2.3. Plant-Based Egg Simulation Products

An increasing interest in the development of egg alternatives is driven by various factors such as consumer preference, allergen reduction, improved nutrition, etc. [33]. Rondoni et al. (2021) used concept mapping and semantic network analysis to explore consumers’ perceptions of plant-based eggs [34]. Their findings show that consumers from the UK and Italy most often associated “price”, “sustainability”, “use”, and “taste” with plant-based eggs. For most respondents, there was an optimistic and positive mindset towards plant-based analogue eggs. As a new food in the food industry, egg simulation products use plant-based elements as the main raw materials to simulate natural eggs in terms of taste, appearance, and organizational structure. The egg white is a liquid substance made from a combination of several ingredients and the yolk part is shaped using a molecular process to combine proprietary ingredients. In addition, since they are not real eggs (in terms of nutrition), these “plant eggs” are naturally free of egg-related pathogens such as salmonella. They contain no cholesterol compared with regular eggs, so for some people who need to keep check on their cholesterol intake the plant egg can somehow be used as a substitute for eggs. Plant-based eggs are mainly constructed by emulsion and gel methods. Therefore, plant protein can be used as a good matrix material for the construction of simulated egg liquid. Hedayati et al. (2022) introduced some plant protein biopolymers that can be recombinant with food hydrocolloids to prepare egg substitute with gelling, foaming, film-forming, emulsifying, binding, and thickening properties and can be used as a substitute for eggs in baked goods [35]. Lu et al. (2022) provided a low-cholesterol egg substitute composition comprising plant protein, fat, and a hydrocolloid system (a mixture of hydrocolloid and cross-linking agents) that can be used in fried and scrambled eggs, as well as in omelets, etc. [36]. Lin et al. (2017) used a mixture of soybean protein isolates, xanthan gum, and emulsifiers to prepare an eggless cake suitable for vegetarians; its physicochemical properties were similar to those of the control cake, such as specific volume, texture, and specific gravity [37]. 

From all these egg substitute applications, it is noted that a combination of plant proteins, hydrocolloids or emulsifiers, and other food ingredients is expected to be used in the development of egg substitutes. At present, the development of egg-mimicked products by researchers is mainly based on plant proteins and polysaccharides [38]. Compared with a single-protein gel system, polysaccharide and protein complexes can usually adjust the gel properties more effectively, change the molecular structure of the two, and make the complex of the two have better solubility, emulsifying and foaming properties, gelling properties, and conformational stability [33]. In the food industry, plant proteins and polysaccharides are often used to build structures and building blocks, due to their functional properties of gelling, thickening and stabilizing surfaces, which play a role in food texture, moisture adsorption, fat stabilization, flavor release, etc. Therefore, research is carried out to create more flexible egg analogues of plant protein-based material. This could be achieved by mixing plant proteins with polysaccharides, such as κ-carrageenan and gellan gum [39]. The pH changes and salt ion modifications such as calcium have also been used to improve the structural and rheological properties of plant-based liquid-egg substitutes [40]. The gel of plant-based egg analogue has a very similar appearance, texture, and thermal behavior to that of a real egg gel, such as the viscosity, gelling temperature, and gel strength [36]. Plant-based egg-analogue gels are more brittle and less chewy and resilient than egg, which may alter their cookability, functional properties, and sensory attributes [41]. In addition, plant protein components contain pigments (polyphenols) that cause an undesirable brown color in plant-based egg analogues. These pigments can be removed using suitable processing operations, which means that this problem can be overcome by producing a pure plant protein ingredient. Current research on plant-based mimicked eggs has focused on nutrition, taste, and appearance; however, not much research has focused on their flavor and aroma [42]. 

### 2.4. Plant-Based Protein Emulsion Foods

Due to “clean labelling” requirements, there is an increasing demand for the development of plant proteins as natural food emulsifiers that are comparable to the emulsifiers traditionally used, such as phospholipids, fatty acid glycerol monoesters and their derivatives, fatty acid esters of sucrose, and fatty acid esters of sorbitan and their derivatives [43]. There is increasing interest in a detailed and systematic comparison of the emulsification properties for plant proteins, leading to a shift towards a reduction in the use of animal proteins in food manufacturing processes. Currently, the preparation of nanoparticles or microgels from plant proteins for stabilizing high internal-phase emulsions (HIPEs) has become a new direction for plant protein applications [44]. However, plant protein emulsions have problems such as difficulties in scaling up production and poor emulsion stability. Based on this, plant protein microemulsion systems can be constructed on a scale according to the mechanism of emulsifying ability regulation. In addition, a recent study has proposed that the long-term preservation of protein-based emulsions can be solved through targeted covalent cross-linking emulsion stability strategies [45]. Highly stable emulsions can be used as carriers for new food ingredients to develop foods that are rich in lipid-soluble functional ingredients and can be dispersed in aqueous-phase food systems for applications in 3D printing and new emulsion or beverage product development [46]. Plant-based protein emulsion foods have gained consumer acceptance, such as ice cream, cream, butter, mayonnaise, and salad dressings. Mayonnaise, one of the most widely used sauces or condiments worldwide, is a typical O/W emulsion, with plant oil as the oil phase and egg yolk, vinegar, cane sugar, salt, mustard, and other additives as the aqueous phase, containing 70–80% fat. Egg yolk plays a key role in the stabilization of mayonnaise as an emulsifier, with lecithin, high-density lipoprotein (HDL), low-density lipoprotein (LDL), and vitellin playing a key emulsifying role. With the rising health consciousness of global consumers and the food market demand towards low-sugar, low-fat, and low-calorie products, the consumption of mayonnaise has been restricted by some people. Therefore, the development of egg yolk alternatives to prepare new egg-free mayonnaise with low cholesterol seems to be in demand. A great deal of research has been conducted to explore the use of plant proteins as a substitute for egg yolks in the preparation of low-cholesterol mayonnaise-like emulsions [47]. Current research and development for plant-based mayonnaise emulsions is focused on three main areas: (1) development of plant protein as an emulsifier to partially replace egg yolk to produce low-cholesterol mayonnaise-like emulsions; (2) development of a low-cholesterol mayonnaise-like emulsion using plant protein as an emulsifier in place of egg yolk; (3) an emulsion with similar physical and chemical properties to mayonnaise prepared from plant proteins to replace mayonnaise.

Mayonnaise has a high proportion of internal phases; the droplets forming the emulsion have a small particle size, allowing a more viscous texture while exhibiting viscoelastic rheological properties with yield stress. At present, some of the studies on the preparation of mayonnaise-like emulsions from alternative egg yolks mostly use various types of plant proteins in combination with polysaccharides or complex polymers as emulsifiers or add polysaccharide thickeners to assist in achieving the rheological properties and stability of mayonnaise. Nikzade et al. (2012) prepared mayonnaise-like emulsions using soy milk and other stabilizers (xanthan gum, guar gum, etc.), replacing all egg yolks [48]. With the increasing additional proportion of xanthan gum and guar gum, the thermal stability, viscosity, hardness, and adhesion of the mayonnaise-like emulsions were increased and the fluid behavior was decreased. Similar to the application of a partial substitute for egg yolk and to achieve similar texture and structural characteristics to mayonnaise, hydrophilic colloids and protein particles with Pickering effects have become the best choice to prepare mayonnaise-like emulsions. The addition of NaCl and sucrose to the pea protein isolate provided an edible mayonnaise-like Pickering emulsion, which increased the particle size with the addition of NaCl and sucrose, resulting in better rheological and textural properties due to the reduction in the distance between droplets [49]. Ruan et al. (2019) prepared HIPEs using citrus fibers and maize peptides, which have superior sensory properties and lower frictional properties, thermal stability, and thixotropic recovery compared with mayonnaise, whereas the maize peptides and soluble functional fibers help to build the interfacial structure of the emulsion and inhibit fat uplift of the emulsion [50]. In addition, O/W HIPEs (ϕ = 0.75) prepared by emulsification–evaporation of wheat gliadin from plant proteins can also be used as a mayonnaise substitute [51]. The resulting HIPEs have similar emulsion droplet sizes, rheological properties, better thixotropic recovery, and thermal stability as mayonnaise. In addition, He et al. (2019) introduced a new rheological additive extracted from plant-based chickpea, which can be developed as a partial substitute for egg yolk to produce mayonnaise-like emulsion with good viscoelastic properties [52].

## 3. Technologies used for Improving the Functional Properties of Plant-Based Proteins

### 3.1. Protein–Protein, Protein–Polysaccharide, Protein–Other Component Interactions

**Figure 2:**
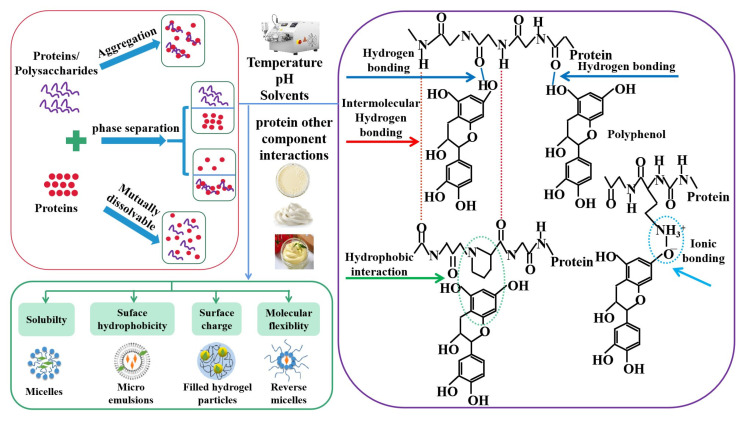
The improvement of functional properties for plant-based proteins to prepare an alternative to animal protein by interaction including protein–protein, protein–polysaccharide, protein–polyphenol, and other components. HIPPEs are high internal-phase Pickering emulsions.

The functional properties of proteins depend mainly on their source, type, structure, and ability to interact with a number of other nutrients. The physicochemical and functional properties of proteins determine the quality of the final product during processing. Therefore, the use of plant-based proteins in the animal protein replacement industry is limited due to some properties. However, studies have shown that the complexation of proteins with other compounds can improve the nutritional and functional properties of plant proteins, such as gelation, emulsification, and solubility. The structural versatility and amphiphilic nature of proteins allow them to interact with several compounds in food under certain conditions; these interactions occur mainly through hydrogen bonding, electrostatic interactions, and hydrophobic and disulfide bonding (Figure 2). Concentration, temperature, ratio, ionic strength, and pH can facilitate the binding of molecular forces on the protein and the folding of the protein. These hybrid protein systems have innovative potential in terms of synergistic technological functionality. Protein–protein interactions (PPI) are considered to be one of the best techniques for enhancing the functional properties of plant proteins, driven by the development of structural and colloidal building blocks. PPI has been widely used to design new structural proteins with improved functional properties, thus expanding the application of plant proteins as alternatives to animal proteins. Ozturk et al. (2022) reported the dispersion of zein protein into pea protein by adding alkaline agents, among which the mixture can be prepared for plant-based meat analogues with better viscoelastic and cohesive properties compared with a single protein, indicating the interaction of the two proteins [53]. Liang et al. (2016) investigated the effect of different globular protein sources (soy, pea, and whey protein) and the ratio of casein to globular protein (6:4 and 4:6) on the stability of complex protein emulsions. It was found that the casein–globulin complex emulsions containing whey protein were less stable than those containing plant globulins [54]. Yerramilli et al. (2017) further investigated the stability mechanism of composite protein emulsions prepared from pea protein and sodium caseinate (1:1). The results showed that the unabsorbed protein mixture in the continuous phase may have inhibited phase separation and aggregation in the composite emulsion; therefore, the composite double protein emulsion was stable for six months (D32 < 200 nm). The pea proteins, with reduced particle size and increased surface hydrophobicity after high-pressure homogenization, could be coated on the surface by the flexible sodium caseinate to prevent pea protein aggregation and achieve the stabilization of the composite emulsion. In a two-protein emulsion system, plant proteins can form a synergistic stable emulsion with animal proteins [55]. The salt-extracted pea protein and whey protein composite gel (2:8, *v*/*v*) prepared by Wong et al. (2013) showed a synergistic enhancement of gel strength in the hot gel [56]. To clarify the interaction between the two protein mixtures, Chihi et al. (2018) investigated different thermal aggregates of β-lactoglobulin and pea globulin; two proteins were heated simultaneously to form mixed thermal aggregates and a mixture of thermal aggregates. It was found that the elasticity and water-holding capacity of the gels for mixed protein were superior to those of the thermo-aggregated mixture gels [57]. Furthermore, a study by He et al. (2020) reported that mixing wheat gluten protein and soy protein isolate at a mass ratio of 0.5:1 to obtain 1% (*w*/*v*) solution through structural interactions resulted in novel edible hydrocolloids with an increase in solubility of wheat gluten protein from 6 to over 66% [1]. The construction of high-quality plant proteins through protein–protein interactions based on nutritional quantitative-efficiency relationships and precise interactions is currently a hot research topic, which can meet the functional, nutritional, and health needs of consumers.

In addition to PPI, interacting compounds include carbohydrates, polyphenols, and vitamins. Within the food sector, the study of protein–polysaccharide complexes has an important role to play in enriching food nutrition and developing innovative food products. Protein–polysaccharide complexes are the products that improve the functionality of proteins; they have better functionality than their precursors. Proteins are often used as emulsifiers due to their amphiphilic structure; polysaccharides are often used as thickeners and gelling agents due to their rheology-altering properties in food systems. The addition of polysaccharides to protein-based emulsions has been reported to improve the stability of emulsions. It has also been found that polysaccharides electrostatically bind with proteins to form a coagulant [58]. The complex reaction and interaction between the two macromolecules give their complex better properties (solubility, emulsification, emulsion stability, foaming, water retention, thermal stability, antioxidant properties, etc.), so the protein–polysaccharide complex has promising applications for the animal protein replacement regime. In addition, polar polyphenols will enhance the emulsification ability of proteins by modifying them, strengthening their hydrophobicity, and weakening their resistance to strain (Figure 2). Thus, the interaction between polyphenols and proteins will change the protein structure, which in turn affects the amphiphilicity and stability of proteins. This effect is often used in the food industry to improve the functional properties of proteins and to enhance product performance. The combination of proteins with other ingredients not only combines the high-quality properties of the protein with its conjugate but also improves the functional properties of the final product, making it more nutritionally complete.

### 3.2. Fermentation

**Figure 3:**
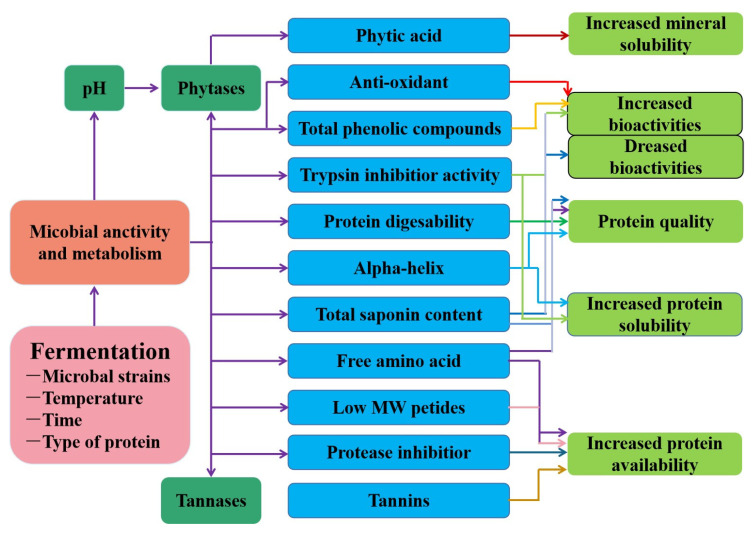
The changes in the composition and functional properties of plant-based proteins treated with fermentation technology.

Fermentation is an ancient food biotechnology that can transform complex organic substances into simple compounds by means of intrinsic organic catalysts produced by microorganisms; it is widely used in the food industry to improve the nutritional, safety, organoleptic, and functional properties of products (Figure 3). Fermented edible plant proteins and their products are mostly studied using inoculated fermentation to obtain a stable and high-quality product (e.g., cheese, yoghurt). Thus, fermentation may be an effective bioprocessing technique for obtaining tasty and hypoallergenic plant protein with good physicochemical properties. *Lactobacillus* and *Bifidobacterium* are the most commonly used bacteria for the fermentation of plant-based products other than *Bacillus subtilis* and fungi (molds). Typically, the protein matrix is linked to various large molecules such as carbohydrates and lipids, as well as small molecules such as phenolic chemicals. During microbial fermentation, microorganisms produce enzymes, such as esterases and amylases, lipases, oligopeptidases, iminopeptidases, and peptide hydrolases, leading to the disruption of complex protein crosslinks accompanied by the changes in the structure and content of nutrients such as free phenolic compounds and carbohydrates, as well as free proteins and amino acids [59]. During fermentation, microorganisms use nutrients to produce proteases that hydrolyze the proteins, increasing their soluble protein content, releasing small peptides and free amino acids, and producing γ-aminobutyric acid. Studies have reported a significant increase in soluble protein and small peptide and amino acid content in the substrate after fermentation. As the protein is hydrolyzed and the amino acids are metabolized and utilized, this results in a reorganization of the amino acids, which further leads to changes in the protein structure.

Yang et al. (2021) found that, during the fermentation process, the proteases produced by the microorganisms reduced the particle size of the soy protein and increased the free amino acid content, changed the structure of the protein, and thus improved the functional properties [60]. Meinlschmidt et al. (2016) studied the effects of liquid fermentation on the physicochemical properties and the sensory and immunoreactivity of soy protein; they found that fermentation increased protein solubility and the ability to bind water and oil, doubled the foaming activity, and reduced immunoreactivity compared with unfermented soy protein [61]. In addition, principal component analysis confirmed a reduction in bitterness and off-flavors in the fermented samples compared with nonfermented soy protein. It was found that fermentation with lactic acid bacteria significantly improved the emulsification of soy protein and increased the water-holding capacity of the protein, resulting in stronger protein gels [62]. Fermentation can effectively degrade antinutritional factors present in plant proteins, such as phytic acid, tannins, and soya globulin. Wang et al. (2022) found that microbial fermentation of soya milk enriched the content of aglycone isoflavones and in situ riboflavin (B_2_) and improved its digestibility and nutritional quality by changing the amino acid composition [63]. Based on the beneficial effects of fermentation, Razavizadeh et al. (2022) prepared meat analogues with better organoleptic and textural properties by fermentation and enzymatic hydrolysis using soybean press cake as a raw material. Fermentation not only affects the structural and functional properties of plant proteins but also promotes their nutritional properties and bioactivity. In addition, fermentation reduces the allergenicity of plant proteins by degrading their allergenic and antinutritional components. Therefore, the application of fermentation technology to plant proteins and their products can confer or improve their processing properties, nutritional characteristics, and bioavailability [64].

### 3.3. Germination

**Figure 4:**
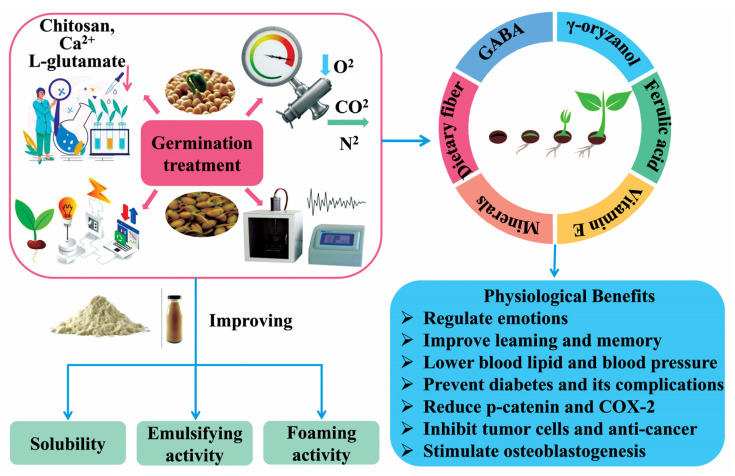
The changes in the composition and functional properties of plant-based proteins treated with germination.

In the field of food science and nutrition, germination is widely used as a major bioprocessing method to enrich the nutritional content of edible seeds, reduce antinutritional factors, increase protein digestibility, and improve functional features (Figure 4) [65].

Germination of cereal and legume seeds causing changes in proteins, amino acids, and other components is an interesting field with great potential. Current research on the germination of pulses and cereals focuses on the nutritional quality and functional activity of the flavonoids and phenolics during seed germination, such as antioxidants and changes in protein fractions and subunits. For example, the crude protein content of *Chenopodium quinoa* and field pea increased after germination, probably due to a lower consumption of its own proteins than the production of new ones, resulting in an increase in the content of various proteins after germination [66]. Concha et al. (2022) investigated the effect of germination on the proteolysis and anti-inflammatory properties of *Pisum sativum* L. grains, showing that storage proteins are hydrolyzed by proteases to release amino acids or small molecule peptides, which synthesize new proteins with small molecule peptides by the oxidation of the carbon backbone after deamination, resulting in an increase in soluble protein content [67]. In addition, soluble proteins contain components that exert anti-inflammatory effects through a variety of mechanisms. Di et al. (2022) studied the effects of different sprouting treatments (0 d, 2 d, and 4 d) on the structural and functional properties of sesame proteins and showed that sprouting significantly increased the in vitro digestibility and solubility of sesame proteins [68]. GABA is a nonprotein amino acid that is widely found in plants and animals that is converted from glutamic acid. It has a variety of effects, including promoting sleep, improving brain function, lowering blood sugar, and lowering blood pressure. Several studies have found that germination leads to a significant accumulation of GABA in cereal and legume seeds [69,70]. These results suggest that germination treatments can improve the structural and functional properties of plant-based proteins and can be an effective way to improve the nutritional properties and processing values of plant seed proteins.

### 3.4. Physical, Chemical, and Enzyme Modification

**Figure 5:**
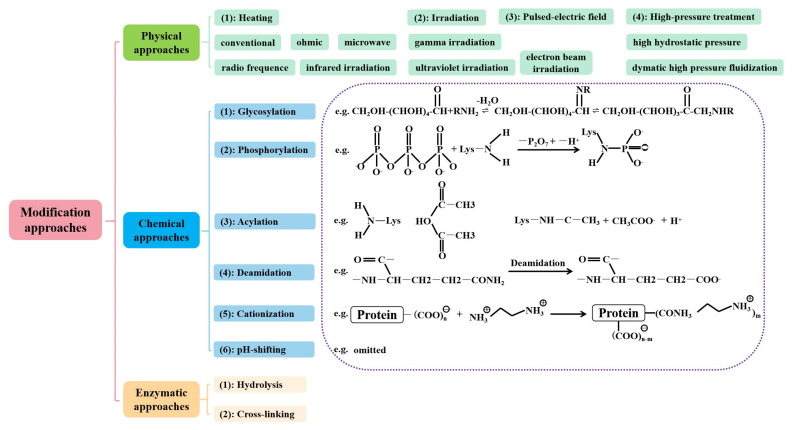
Physical, chemical, and enzymatic methods for the modification of plant-based proteins.

Protein modification refers to the artificial modification of protein structures by suitable methods according to certain specific needs, thus improving the processing properties of the protein [71]. In general, protein modification is commonly accomplished by physical, chemical, and enzymatic methods (Figure 5). Physical modification refers to the alteration of the advanced structure and intermolecular aggregation of proteins by certain physical means, such as ultramicronization, high-frequency electric field, high pressure treatment, ultrasonic treatment, addition of thickening agents, and supercritical CO_2_ fluid treatment, etc. Moderate physical modifications have little effect on the primary structure of the protein. However, it can improve the functional properties of the protein by altering the secondary and tertiary structure. Plant proteins are modified by heat deformation or by adding some edible gum to make the protein gel well and then by high-speed shearing to reduce the protein particle size to obtain plant-based protein emulsion foods [72]. The physical modification method generally does not produce toxic byproducts and it is easy to control and use.

Chemical modification is based on the principle of interaction between chemical reagents and proteins, where the peptide bonds of some proteins are broken or other functional groups are introduced into the protein structure, thereby improving its functional properties. For plant-based protein products, the hydration properties (water-holding, solubility, adhesion, etc.), surface properties (emulsification, foaming, etc.), structural properties (gelation, viscoelasticity, etc.), and organoleptic properties (color, taste, odor, smoothness, palatability, chewiness, etc.) are improved [73]. The enhancement of these functional properties plays an important role in the application of plant proteins. Common modification methods include acylation, phosphorylation, deamidation, glycosylation, and acid-base modification. He et al. (2019) used acetic acid, tartaric acid, and citric acid deamidation combined with heat treatment to modify wheat gluten proteins to obtain three different modified-gluten proteins and then added the three deamidated gluten proteins as substitutes to skimmed-milk powder in ice cream to study their effects on the rheological properties of the mixture and the physical properties [52]. All the mixtures exhibited non-Newtonian shear thinning and a more elastic behavior rather than a viscous structure. The results of the partial least squares discriminant analysis of the physicochemical and rheological properties for the ice cream samples indicated that the citrate-deamidated gluten proteins showed appropriate rheological and physical properties, which could be used for further development as a fat substitute for ice cream. In the preparation of egg-simulation products from plant proteins, the chemically modified proteins are often compounded with gums to improve the rheological properties of the system and thus more closely match the physical properties of the egg [74,75]. Chemical modification has the advantages of short reaction times, low cost, low equipment requirements, and significant modification effects.

Enzymatic modifications are divided into two categories: nonhydrolytic modifications and hydrolytic modifications. Among them, enzymatic hydrolysis modification refers to the process of using specific proteases to break down large molecules into small peptide chains of various chain lengths [76]. It is also the key to the preparation of plant-based protein emulsion foods. At a certain level of hydrolysis, proteins form a reticulated gel that absorbs a large amount of water and has a soft texture, resulting in a smooth viscous-like texture [77]. However, excessive hydrolysis prevents the formation of gels. The main proteases used for enzymatic modification are trypsin, papain, and certain microbial enzymes [78]. Enzymatic modification can significantly enhance the functional properties of proteins such as emulsification, which can make plant-based protein emulsion well-suited for use in foods such as ice cream and mayonnaise [24]. The advantage of enzymatic modification is that the whole process takes place in a mild environment and there is a high degree of specificity, which has been extensively studied and applied in food processing due to its safety [78]. In addition, there are some studies that combine more than two techniques. Li et al. (2021) combined chemical modification with enzymatic modification to produce reaction products of sugar and soy protein hydrolysates using the glycosylation reaction, which performed well in terms of all the functional properties [79].

## 4. Health Benefits of Plant-Based Protein Products

**Figure 6:**
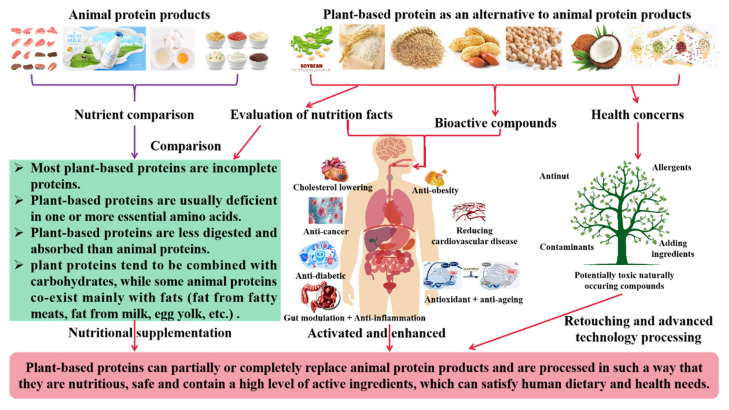
A comparison of the effects of plant versus animal protein diets on chronic disease in humans.

The comparison of the effects of plant versus animal protein diets on chronic disease in humans is shown in Figure 6. For nutritional and safety evaluation of plant-based alternative proteins, most animal-derived proteins have protein digestibility corrected amino acid scores (PDCAAS) above or close to 1.0 and are considered to be a complete protein source, whereas many plant-based proteins are often deficient in one or more essential amino acids and generally have lower PDCAAS scores than animal proteins. In general, there are not many differences between plant and animal proteins for protein supplement, but there are some differences in the composition and quantity of amino acids. Animal proteins are relatively compatible with the nutritional structure of humans, with the type and structure of their proteins more closely resembling the structure and quantity of human proteins and generally containing the nine essential amino acids, especially egg and dairy products. The body produces 11 amino acids, but must obtain the other 9 from food. Animal products are complete proteins, which means they contain all the amino acids [80]. Some plant-based products, such as soya and quinoa, are also complete proteins, while others are incomplete proteins. Although plant protein sources are wide, there is a gap between the variety and relative quantity of plant proteins and the requirements of the human body compared with animal protein, such as the lack of immunoglobulins and certain essential amino acids in plant proteins. The contents of methionine (e.g., beans, nuts, and seeds), lysine (e.g., grains such as wheat) or tryptophan (e.g., maize) in plant proteins are low; However, some nonessential amino acids such as arginine, glycine, alanine, and serine are relatively higher than those in animal proteins. Although the essential amino acid ratios of plant proteins are not as close to those of animal proteins, it is possible to meet the essential amino acid requirements of the human body by consuming sufficient and different plant proteins. Plant proteins are also usually more difficult to digest than animal proteins. Fiber and other components in plants make it harder for digestive enzymes to break down proteins for absorption in our digestive tract [81]. However, by limiting the absorption of carbohydrates or cholesterol, this property of plant-based protein can be beneficial in areas such as heart health or blood-sugar management.

In addition, plant proteins can be made more digestible through soaking, cooking, concentration, or separation techniques. Plant-based protein products are cholesterol-free and lactose-free, so they are not a burden to eat and at the same time meet the body’s protein requirements. In terms of the state of protein presence in food, plant proteins tend to be combined with carbohydrates (non-starch polysaccharides and dietary fiber), while some animal proteins contain relative higher fats, e.g., fatty meats, milk, egg yolk, etc. [2,82,83]. These factors can bring about differences in the nutritional values and health issues of plant and animal proteins. For example, the intakes of some protein from intramuscular fat in pork are sometimes accompanied by a high intake of saturated fat, compared with plant proteins that provide more unsaturated fatty acids [2]. However, the consumption of plant protein (not only in terms of protein) also includes functional ingredients such as dietary fibers, isoflavones, and phospholipids. Compared with animal protein, plant protein has more health benefits, including a reduced risk of cardiovascular disease, type 2 diabetes, and certain cancers. For example, milk protein has been shown to have weight control and triglyceride-lowering effects, while soy protein isolate has a more pronounced lipid-lowering effect than milk protein [84]. Studies have shown that excessive red-meat consumption increases the risk of cardiovascular disease and colorectal cancer. For individual health considerations, the dietary guidelines of some countries recommend not more than 100 g of red meat per day for adults [85]. Huang et al. (2020) suggested that replacing animal protein with plant protein could reduce mortality and cardiovascular disease rates by a full double-digit amount [86]. There was a clear link between increased plant protein intake and lower mortality. The results showed that an increase in dietary protein intake was associated with a reduction in overall mortality, with a 10% reduction in the risk of premature death for both men and women when 3% of the energy provided by animal protein was replaced with plant protein, while the risk of cardiovascular disease fell by 11% and 12% in men and women, respectively. If the 3% was specific to certain foods, it was mainly eggs and red meat that were the most significant, with a 24% and 21% reduction in risk for men and women, respectively, when replacing eggs, and 13% and 15% for red meat. However, biases in this study that may have made the results somewhat controversial included participants with higher plant protein intakes were more likely to have diabetes, a higher educational level, be more physically active, and less likely to be current smokers, etc. In previous studies, it has been found that high quality plant proteins can reduce the risk of cardiovascular disease and breast cancer death in humans [87]. Plant proteins also help the flora in the gut to function better and reduce the potential toxicity of many human metabolites, which are often associated with chronic inflammation and carcinogenic metabolites [88]. The active peptides extracted from plant proteins exert antioxidant, anti-inflammatory, and antihypertensive effects, as well as lower blood cholesterol levels [89]. Dr Campbell, the world’s leading nutritionist, has found that plant-based diets are beneficial in improving and preventing a range of chronic diseases [90]. Plant-based diets can even reverse the course of chronic diseases without the use of drugs and can counteract the effects of some harmful substances on health. The nutritional composition of plant protein products is similar to that of animal protein products, but plant protein products often have a variety of taste and flavor enhancing ingredients added to them, often including flavoring agents, coloring agents, and binders, which may lead to excessive intakes of substances such as salt and sugar [91]. These can cause human health problems, which need to be mitigated or addressed through updates in ingredients, processes, and technology. The most important feature of plant-based products is that they replace animal protein in food and beverages with plant protein, which is more in line with the new generation of consumer’s pursuit of a healthy diet than animal protein products. Although plant protein advantages are better for health, the major difference between plant and animal proteins is the amino acids. Because many amino acids cannot be synthesized by the body itself, it is important for those on a vegetarian diet to diversify their sources of plant protein to avoid nutritional deficiencies.

## 5. Conclusions

In the past years, plant protein has emerged as a sustainable and important nutritional source and the use of plant-based protein products to supplement or replace some of the deficiencies of animal protein has emerged as a hot research topic. As a high-quality protein resource, plant proteins have very good potential for exploration, which plays a more important role in meeting the demand for food proteins. At present, the commercialization of plant-based protein products as an alternative to animal protein is facing problems such as small ranges of available products and bottlenecks in processing technology. In addition, the functional properties of plant-based proteins are poorly compared with those of animal proteins, particularly in terms of solubility in water and stability and ability to foam. These plant-based proteins are reported to have good functional properties following some modifications and techniques, including physical, chemical, enzyme, fermentation, and germination. Mixed proteins or other components, fermentation, and germination have been found to be the main reconstruction techniques for creating protein complexes with nutritional and functional properties. There are some challenges to address some of the technical barriers of plant-based proteins, particularly solubility and nutritional properties. Plant proteins should be investigated more as alternatives to animal proteins, especially the combination of two or more technologies, such as protein interaction technology (to enhance functional properties) and fermentation or germination technology (to enhance nutritional properties), to produce new plant protein complexes with enhanced properties. Compared with animal proteins, plant proteins have a unique role in human health against cardiovascular disease, type 2 diabetes, and certain tumors. The only areas of concern are the presence of allergens and off-flavor components of plant protein-based foods, which affect the flavor and safety of plant protein products and limit their widespread use in the food sector. There is an urgent need to increase the direct use of plant-based proteins in the human diet and to modify the functional and organoleptic properties of proteins through various processing processes. Moreover, plant-based alternative proteins face great challenges in terms of low cost and efficient preparation, precise control of processing, nutritional and safety evaluation, and flavor and texture regulation; therefore, more emphasis is needed to accelerate their industrial application by advancing in process technology and new approaches. Future research in the field of plant proteins can be summarized in two basic strategies, namely protein fortification and complementation. Although much has been achieved in various processing technologies for plant proteins, there is a need to develop fully effective processing methods (precise and efficient) to exploit proteins with high nutritional value and functional properties and thus hasten the further industrialization of plant proteins.

## References

[1] He J., Wang R., Feng W., Chen Z.X., Wang T. (2020). Design of novel edible hydrocolloids by structural interplays between wheat gluten proteins and soy protein isolates. Food Hydrocoll..

[2] Lappi J., Silventoinen-Veijalainen P., Vanhatalo S., Rosa-Sibakov N., Sozer N. (2022). The nutritional quality of animal-alternative processed foods based on plant or microbial proteins and the role of the food matrix. Trends Food Sci. Tech..

[3] Basak S., Banerjee A., Pathak S., Duttaroy A.K. (2022). Dietary fats and the gut microbiota: Their impacts on lipid-induced metabolic syndrome. J. Funct. Foods.

[4] de las Heras-Delgado S., Shyam S., Cunillera È., Dragusan N., Salas-Salvadó J., Babio N. (2023). Are plant-based alternatives healthier? A two-dimensional evaluation from nutritional and processing standpoints. Food Res. Int..

[5] Lauk C., Kaufmann L., Theurl M.C., Wittmann F., Eder M., Hortenhuber S., Freyer B., Krausmann F. (2022). Demand side options to reduce greenhouse gas emissions and the land footprint of urban food systems: A scenario analysis for the City of Vienna. J. Clean. Prod..

[6] Alae-Carew C., Green R., Stewart C., Cook B., Dangour A.D., Scheelbeek P.F.D. (2022). The role of plant-based alternative foods in sustainable and healthy food systems: Consumption trends in the UK. Sci. Total Environ..

[7] Qi X.X., Shen P. (2020). Associations of dietary protein intake with all-cause, cardiovascular disease, and cancer mortality: A systematic review and meta-analysis of cohort studies. Nutr. Metab. Cardiovasc. Dis..

[8] Cai J.S., Feng J.Y., Ni Z.J., Ma R.H., Thakur K., Wang S.Y., Hu F., Zhang J.G., Wei Z.J. (2021). An update on the nutritional, functional, sensory characteristics of soy products, and applications of new processing strategies. Trends Food Sci. Tech..

[9] Malek L., Umberger W.J. (2023). Protein source matters: Understanding consumer segments with distinct preferences for alternative proteins. Future Foods..

[10] Shaghaghian S., McClements D.J., Khalesi M., Garcia-Vaquero M., Mirzapour-Kouhdasht A. (2022). Digestibility and bioavailability of plant-based proteins intended for use in meat analogues: A review. Trends Food Sci. Tech..

[11] Otero D.M., Mendes G.D.L., Lucas A.J.D., Christ-Ribeiro A., Ribeiro C.D.F. (2022). Exploring alternative protein sources: Evidence from patents and articles focusing on food markets. Food Chem..

[12] Sogari G., Caputo V., Petterson A.J., Mora C., Boukid F. (2023). A Sensory Study on Consumer Valuation for Plant-Based Meat Alternatives: What is liked and disliked the most?. Food Res. Int..

[13] Meng A., Chen F., Zhao D., Wei Y., Zhang B. (2022). Identifying changes in soybean protein properties during high-moisture extrusion processing using dead-stop operation. Food Chem..

[14] Wang Y., Lyu B., Fu H., Li J., Ji L., Gong H., Zhang R., Liu J., Yu H. (2023). The development process of plant-based meat alternatives: Raw material formulations and processing strategies. Food Res. Int..

[15] Xie Y.T., Cai L.L., Zhao D., Liu H., Xu X.L., Zhou G.H., Li C.B. (2022). Real meat and plant-based meat analogues have different in vitro protein digestibility properties. Food Chem..

[16] Zhang T., Dou W., Zhang X., Zhao Y., Zhang Y., Jiang L., Sui X. (2021). The development history and recent updates on soy protein-based meat alternatives. Trends Food Sci. Tech..

[17] Xiang N., Jr J., Stout A.J., Rubio N.R., Chen Y., Kaplan D.L. (2022). 3D porous scaffolds from wheat glutenin for cultured meat applications. Biomaterials.

[18] Hu F., Zou P.R., Zhang F., Thakur K., Khan M.R., Busquets R., Zhang J.G., Wei Z.J. (2022). Wheat gluten proteins phosphorylated with sodium tripolyphosphate, changes in structure to improve functional properties for expanding applications. Curr. Res. Food Sci..

[19] Zou P.R., Hu F., Ni Z.J., Zhang F., Thakur K., Zhang J.G., Wei Z.J. (2022). Effects of phosphorylation pretreatment and subsequent transglutaminase cross-linking on physicochemical, structural, and gel properties of wheat gluten. Food Chem..

[20] Zou P.R., Hu F., Zhang F., Thakur K., Rizwan Khan M., Busquets R., Zhang J.G., Wei Z.J. (2022). Hydrophilic co-assembly of wheat gluten proteins and wheat bran cellulose improving the bioavailability of curcumin. Food Chem..

[21] Chiang J.H., Loveday S.M., Hardacre A.K., Parker M.E. (2019). Effects of soy protein to wheat gluten ratio on the physicochemical properties of extruded meat analogues. Food Struct-Neth..

[22] Yuliarti O., Kovis T.J.K., Yi N.J. (2021). Structuring the meat analogue by using plant-based derived composites. J. Food Eng..

[23] Sajib M., Forghani B., Vate K.N., Abdollahi M. (2023). Combined effects of isolation temperature and pH on functionality and beany flavor of pea protein isolates for meat analogue applications. Food Chem..

[24] Kumar M., Tomar M., Punia S., Dhakane-Lad J., Dhumal S., Changan S., Senapathy M., Berwal M.K., Sampathrajan V., Sayed A.A.S. (2022). Plant-based proteins and their multifaceted industrial applications. LWT-Food Sci. Technol..

[25] Zhang X., Zhao Y., Zhang T.Y., Zhang Y., Jiang L.Z., Sui X.A. (2022). High moisture extrusion of soy protein and wheat gluten blend: An underlying mechanism for the formation of fibrous structures. LWT-Food Scie. Technol..

[26] Silva A.R.A., Silva M.M.N., Ribeiro B.D. (2020). Health issues and technological aspects of plant-based alternative milk. Food Res. Int..

[27] Aydar E.F., Tutuncu S., Ozcelik B. (2020). Plant-based milk substitutes: Bioactive compounds, conventional and novel processes, bioavailability studies, and health effects. J. Funct. Foods.

[28] Boukid F., Hassoun A., Zouari A., Tülbek M.Ç., Mefleh M., Aït-Kaddour A., Castellari M. (2023). Fermentation for Designing Innovative Plant-Based Meat and Dairy Alternatives. Foods.

[29] Vogelsang-O’Dwyer M., Zannini E., Arendt E.K. (2021). Production of pulse protein ingredients and their application in plant-based milk alternatives. Trends Food Sci. Tech..

[30] Sarangapany A.K., Murugesan A., Annamalai A.S., Balasubramanian A., Shanmugam A. (2022). An overview on ultrasonically treated plant-based milk and its properties—A Review. Appl. Food Res..

[31] Bocker R., Silva E.K. (2022). Innovative technologies for manufacturing plant-based non-dairy alternative milk and their impact on nutritional, sensory and safety aspects. Future Foods.

[32] Salve A.R., Pegu K., Arya S.S. (2019). Comparative assessment of high-intensity ultrasound and hydrodynamic cavitation processing on physico-chemical properties and microbial inactivation of peanut milk. Ultrason. Sonochem..

[33] Lu Z., Lee P.R., Yang H. (2022). Chickpea flour and soy protein isolate interacted with κ-carrageenan via electrostatic interactions to form egg omelets analogue. Food Hydrocoll..

[34] Rondoni A., Grebitus C., Millan E., Asioli D. (2021). Exploring consumers’ perceptions of plant-based eggs using concept mapping and semantic network analysis. Food Qual. Prefer..

[35] Hedayati S., Jafari S.M., Babajafari S., Niakousari M., Mazloomi S.M. (2022). Different food hydrocolloids and biopolymers as egg replacers: A review of their influences on the batter and cake quality. Food Hydrocoll..

[36] Lu Z., Liu Y., Yi E., Jayne L., Andrew C., Lee P.R., Yang H. (2022). Effect of starch addition on the physicochemical properties, molecular interactions, structures, and in vitro digestibility of the plant-based egg analogues. Food Hydrocoll..

[37] Lin M.Y., Tay S.H., Yang H.S., Yang B., Li H.L. (2017). Replacement of eggs with soybean protein isolates and polysaccharides to prepare yellow cakes suitable for vegetarians. Food Chem..

[38] Murray B.S. (2020). Recent Developments in Food Foams. Curr. Opin. Colloid Interface Sci..

[39] McClements D.J., Grossmann L. (2021). A brief review of the science behind the design of healthy and sustainable plant-based foods. Npj Sci. Food.

[40] Wang Y., Zhao J., Zhang S.C., Zhao X.Z., Liu Y.F., Jiang J., Xiong Y.L. (2022). Structural and rheological properties of mung bean protein emulsion as a liquid egg substitute: The effect of pH shifting and calcium. Food Hydrocoll..

[41] Zhou H., Vu G., McClements D.J. (2022). Formulation and characterization of plant-based egg white analogs using RuBisCO protein. Food Chem..

[42] Soni M., Maurya A., Das S., Prasad J., Yadav A., Singh V.K., Singh B.K., Dubey N.K., Dwivedy A.K. (2022). Nanoencapsulation strategies for improving nutritional functionality, safety and delivery of plant-based foods: Recent updates and future opportunities. Plant Nano Biol..

[43] Lingiardi N., Galante M., de Sanctis M., Spelzini D. (2022). Are quinoa proteins a promising alternative to be applied in plant-based emulsion gel formulation?. Food Chem..

[44] Su J., Ma Q., Cai Y., Li H., Yuan F., Ren F., Wang P., der Meeren V.P. (2023). Incorporating surfactants within protein-polysaccharide hybrid particles for high internal phase emulsions (HIPEs): Toward plant-based mayonnaise. Food Hydrocoll..

[45] Yan S., Yao Y., Xie X., Zhang S., Huang Y., Zhu H., Li Y., Qi B. (2022). Comparison of the physical stabilities and oxidation of lipids and proteins in natural and polyphenol-modified soybean protein isolate-stabilized emulsions. Food Res. Int..

[46] An Z., Liu Z., Mo H., Hu L., Li H., Xu D., Chitrakar B. (2023). Preparation of Pickering emulsion gel stabilized by tea residue protein/xanthan gum particles and its application in 3D printing. J. Food Eng..

[47] Ribeiro A., Lopes J.C.B., Dias M.M., Barreiro M. (2023). Pickering Emulsions Based in Inorganic Solid Particles: From Product Development to Food Applications. Molecules..

[48] Nikzade V., Tehrani M.M., Saadatmand-Tarzjan M. (2012). Optimization of low-cholesterol-low-fat mayonnaise formulation: Effect of using soy milk and some stabilizer by a mixture design approach. Food Hydrocoll..

[49] Li S.S., Jiao B., Meng S., Fu W.M., Faisal S., Li X.M., Liu H.Z., Wang Q. (2022). Edible mayonnaise-like Pickering emulsion stabilized by pea protein isolate microgels: Effect of food ingredients in commercial mayonnaise recipe. Food Chem..

[50] Ruan Q.J., Yang X.Q., Zeng L.H., Qi J.R. (2019). Physical and tribological properties of high internal phase emulsions based on citrus fibers and corn peptides. Food Hydrocoll..

[51] Liu X., Guo J., Wan Z.L., Liu Y.Y., Ruan Q.J., Yang X.Q. (2018). Wheat gluten-stabilized high internal phase emulsions as mayonnaise replacers. Food Hydrocoll..

[52] He W.M., Zhao W., Yang R.J. (2019). Effects of wheat gluten modified by deamidation-heating with three different acids on the microstructure of model oil-in-water emulsion and rheological-physical property of ice cream. Food Hydrocoll..

[53] Ozturk O.K., Salgado A.M., Holding D.R., Campanella O.H., Hamaker B.R. (2022). Dispersion of zein into pea protein with alkaline agents imparts cohesive and viscoelastic properties for plant-based food analogues. Food Hydrocoll..

[54] Liang Y.C., Wong S.S., Pham S.Q., Tan J.J. (2016). Effects of globular protein type and concentration on the physical properties and flow behaviors of oil-in-water emulsions stabilized by micellar casein-globular protein mixtures. Food Hydrocoll..

[55] Yerramilli M., Longmore N., Ghosh S. (2017). Improved stabilization of nanoemulsions by partial replacement of sodium caseinate with pea protein isolate. Food Hydrocoll..

[56] Wong D., Vasanthan T., Ozimek L. (2013). Synergistic enhancement in the co-gelation of salt-soluble pea proteins and whey proteins. Food Chem..

[57] Chihi M.L., Sok N., Saurel R. (2018). Acid gelation of mixed thermal aggregates of pea globulins and beta-lactoglobulin. Food Hydrocoll..

[58] Sarkar A., Dickinson E. (2020). Sustainable food-grade Pickering emulsions stabilized by plant-based particles. Curr. Opin. Colloid..

[59] Sharma R., Garg P., Kumar P., Bhatia S.K., Kulshrestha S. (2020). Microbial Fermentation and Its Role in Quality Improvement of Fermented Foods. Fermentation.

[60] Yang X.Y., Ke C.X., Li L. (2021). Physicochemical, rheological and digestive characteristics of soy protein isolate gel induced by lactic acid bacteria. J. Food Eng..

[61] Meinlschmidt P., Ueberham E., Lehmann J., Schweiggert-Weisz U., Eisner P. (2016). Immunoreactivity, sensory and physicochemical properties of fermented soy protein isolate. Food Chem..

[62] Ren Y.M., Li L. (2022). The influence of protease hydrolysis of lactic acid bacteria on the fermentation induced soybean protein gel: Protein molecule, peptides and amino acids. Food Res. Int..

[63] Wang R., Thakur K., Feng J.Y., Zhu Y.Y., Zhang F., Russo P., Spano G., Zhang J.G., Wei Z.J. (2022). Functionalization of soy residue (okara) by enzymatic hydrolysis and LAB fermentation for B-2 bio-enrichment and improved in vitro digestion. Food Chem..

[64] Razavizadeh S., Alencikiene G., Vaiciulyte-Funk L., Ertbjerg P., Salaseviciene A. (2022). Utilization of fermented and enzymatically hydrolyzed soy press cake as ingredient for meat analogues. LWT-Food Sci. Technol..

[65] Liu M., Childs M., Loos M., Taylor A., Smart L.B., Abbaspourrad A. (2022). The effects of germination on the composition and functional properties of hemp seed protein isolate. Food Hydrocoll..

[66] Pilco-Quesada S., Tian Y., Yang B.R., Repo-Carrasco-Valencia R., Suomela J.P. (2020). Effects of germination and kilning on the phenolic compounds and nutritional properties of quinoa (*Chenopodium quinoa*) and kiwicha (*Amaranthus caudatus*). J. Cereal Sci..

[67] Concha D.D.M., Martinez J.E.B., Velazquez T.G.G., Martinez C.J., Ruiz J.C.R. (2022). Impact of germination time on protein solubility and anti-inflammatory properties of Pisum sativum L grains. Food Chem. X.

[68] Di Y., Li X., Chang X.W., Gu R.J., Duan X., Liu F.G., Liu X.B., Wang Y.T. (2022). Impact of germination on structural, functional properties and in vitro protein digestibility of sesame (*Sesamum indicum* L.) protein. LWT-Food Sci. Technol..

[69] Huang G.C., Cai W.X., Xu B.J. (2017). Improvement in beta-carotene, vitamin B-2, GABA, free amino acids and isoflavones in yellow and black soybeans upon germination. LWT-Food Sci. Technol..

[70] Sharma S., Saxena D.C., Riar C.S. (2018). Changes in the GABA and polyphenols contents of foxtail millet on germination and their relationship with in vitro antioxidant activity. Food Chem..

[71] Muhoza B., Qi B.K., Harindintwali J.D., Koko M.Y.F., Zhang S., Li Y. (2022). Combined plant protein modification and complex coacervation as a sustainable strategy to produce coacervates encapsulating bioactives. Food Hydrocoll..

[72] Nowacka M., Trusinska M., Chraniuk P., Drudi F., Lukasiewicz J., Nguyen N.P., Przybyszewska A., Pobiega K., Tappi S., Tylewicz U. (2023). Developments in plant proteins production for meat and fish analogues. Molecules..

[73] Wang R.C., Guo S.T. (2021). Phytic acid and its interactions: Contributions to protein functionality, food processing, and safety. Compr. Rev. Food Sci. Food Saf..

[74] Zha F.C., Yang Z.Y., Rao J.J., Chen B.C. (2021). Gum arabic-mediated synthesis of glyco-pea protein hydrolysate via maillard reaction improves solubility, flavor profile, and functionality of plant protein. J. Agr. Food Chem..

[75] Wei Q.J., Nie P., Gong J.T., Wei C.K., Thakur K., Hu F., Wei Z.J. (2022). Antibacterial and food preservative attributes of maillard reaction products of shrimp shell chitosan. Curr. Top. Nutraceut. Res..

[76] Brückner-Gühmann M., Kratzsch A., Sozer N., Drusch S. (2021). Oat protein as plant-derived gelling agent: Properties and potential of modification. Future Foods..

[77] Shen Y.T., Hong S., Singh G., Koppel K., Li Y.H. (2022). Improving functional properties of pea protein through “green” modifications using enzymes and polysaccharides. Food Chem..

[78] Nasrabadi M.N., Doost A.S., Mezzenga R. (2021). Modification approaches of plant-based proteins to improve their techno-functionality and use in food products. Food Hydrocoll..

[79] Li L., He H., Wu D., Lin D., Qin W., Meng D., Yang R., Zhang Q. (2021). Rheological and textural properties of acid-induced soybean protein isolate gel in the presence of soybean protein isolate hydrolysates or their glycosylated products. Food Chem..

[80] Accardo F., Miguens-Gomez A., Lolli V., Faccini A., Ardevol A., Terra X., Caligiani A., Pinent M., Sforza S. (2022). Molecular composition of lipid and protein fraction of almond, beef and lesser mealworm after in vitro simulated gastrointestinal digestion and correlation with the hormone-stimulating properties of the digesta. Food Res. Int..

[81] Opazo-Navarrete M., Freire D.T., Boom R.M., Janssen A.E.M. (2019). The influence of starch and fibre on In Vitro protein digestibility of dry fractionated quinoa seed (Riobamba variety). Food Biophys..

[82] Gastaldello A., Giampieri F., De Giuseppe R., Grosso G., Baroni L., Battino M. (2022). The rise of processed meat alternatives: A narrative review of the manufacturing, composition, nutritional profile and health effects of newer sources of protein, and their place in healthier diets. Trends Food Sci. Tech..

[83] Bryant C.J. (2022). Plant-based animal product alternatives are healthier and more environmentally sustainable than animal products. Future Foods.

[84] Shi W., Hou T., Guo D.J., He H. (2019). Evaluation of hypolipidemic peptide (Val-Phe-Val-Arg-Asn) virtual screened from chickpea peptides by pharmacophore model in high-fat diet-induced obese rat. J. Funct. Foods.

[85] Clare K., Maani N., Milner J. (2022). Meat, money and messaging, How the environmental and health harms of red and processed meat consumption are framed by the meat industry. Food Policy.

[86] Huang J.Q., Liao L.M., Weinstein S.J., Sinha R., Graubard B.I., Albanes D. (2020). Association Between Plant and Animal Protein Intake and Overall and Cause-Specific Mortality. JAMA Inter. Med..

[87] Qin P., Wang T., Luo Y. (2022). A review on plant-based proteins from soybean: Health benefits and soy product development. J. Agric. Food Res..

[88] Saeed M., Shoaib A., Kandimalla R., Javed S., Almatroudi A., Gupta R., Aqil F. (2022). Microbe-based therapies for colorectal cancer: Advantages and limitations. Semin. Cancer Biol..

[89] Durand E., Beaubier S., Ilic I., Fine F., Kapel R., Villeneuve P. (2021). Production and antioxidant capacity of bioactive peptides from plant biomass to counteract lipid oxidation. Curr. Res. Food Sci..

[90] Kim J., Kim H., Giovannucci E.L. (2021). Plant-based diet quality and the risk of total and disease-specific mortality, A population-based prospective study. Clin. Nutr..

[91] Curtain F., Grafenauer S. (2019). Plant-Based Meat Substitutes in the Flexitarian Age, An Audit of Products on Supermarket Shelves. Nutrients.

